# Over-the-counter sale of antibiotics during COVID-19 outbreak by community pharmacies in Saudi Arabia: a simulated client study

**DOI:** 10.1186/s12913-022-07553-x

**Published:** 2022-01-29

**Authors:** Hani M. J. Khojah

**Affiliations:** Department of Clinical and Hospital Pharmacy, College of Pharmacy, Taibah University, P. O. Box 30051, Madinah, 41477 Kingdom of Saudi Arabia

**Keywords:** Antibiotic, Resistance, Misuse, COVID-19, Over-the-counter, Community pharmacy, Saudi Arabia

## Abstract

**Background:**

Recent studies have reflected increased global concern regarding the possible acceleration of bacterial resistance secondary to the reported overuse and misuse of antibiotics during the COVID-19 pandemic. Therefore, this study aimed to investigate the nonprescribed sale of antibiotics by community pharmacies in Saudi Arabia during the COVID-19 outbreak and the pharmacists’ skills in triaging COVID-19 suspects.

**Methods:**

Herein, 120 pharmacies were randomly selected and surveyed by simulated clients who presented gradual demands to convince the encountered pharmacists to agree to the over-the-counter sale of antibiotics. The pharmacists’ responses and counseling skills were documented in addition to their effectiveness in triaging suspected COVID-19 cases.

**Results:**

Nineteen pharmacists (15.8%) were convinced to sell nonprescribed antibiotics after various levels of demand by clients. Moreover, twenty pharmacists (16.7%), who refused to sell nonprescribed antibiotics, referred the clients to clinics where they could easily obtain prescriptions, or to other pharmacies that violate the system. In addition, 29 pharmacists (24.2%) were not concerned about possible COVID-19 suspects, and 47–66 (39.2–55%) of them demonstrated different responses and recommendations. Moreover, 12 pharmacists of the 19 who violated the law did not offer any counseling regarding the use of the antibiotics.

**Conclusions:**

The nonprescribed sale of antibiotics is still prevalent and may have increased during the COVID-19 outbreak in Saudi Arabia, thereby increasing the risk of accelerated bacterial resistance. The pharmacists’ skills in triaging COVID-19 suspects and patient education and counseling were below expectations. Further follow-up studies are highly recommended.

## Background

Antibiotics include a wide range of compounds prescribed primarily for treating various bacterial infections in humans as well as animals [1]. Unfortunately, many species of bacteria develop immunity against antibiotics through several mechanisms, making these bacteria resistant to the effect of these lifesaving compounds [2]. Bacterial resistance may occur following repeated exposure to the antibiotic or when the con-centration of the antibiotic is not enough to kill or suppress the multiplication of these microorganisms [3]. This often happens when antibiotics are misused or taken for a shorter period than prescribed [4, 5]. A common example of multi-antibiotic bacterial resistance is the methicillin-resistant *Staphylococcus aureus* (MRSA) that resists several groups of antibiotics (including fluoroquinolones, macrolides, lincosamides, tetracyclines, and aminoglycosides) and causes serious infections such as pyogenic endocarditis, pyogenic skin and soft tissue infections, otitis media, septic arthritis, suppurative pneumonia, and osteomyelitis [6].

The misuse of antibiotics was and still is a concern for health institutions around the world due to its disastrous consequences and the paucity of effective antibiotics against certain infections [7]. Moreover, bacterial resistance to antimicrobials confuses healthcare providers and places an economic burden on health systems because it limits the choices of alternative, effective agents, which are also more expensive, and increases hospital admissions [8, 9]. For these reasons, international health institutions and authorities have issued strict guidelines to regulate the prescription, dispensing, and sale of antibiotics, especially in community pharmacies [10, 11]. In spite of this, malpractice in selling antibiotics without a regular prescription is still rampant in many countries [12–15].

The overuse and misuse of antibiotics during the COVID-19 pandemic takes on another dimension. This global, highly contagious, and deadly viral infection is caused by the novel coronavirus, SARS-CoV-2, that originated in China in December 2019 [16], and struck Saudi Arabia in March 2020 [17]. By January 2021, the time this manuscript was written, the pandemic had afflicted around 20.5 million people (296,000 from Saudi Arabia) and claimed the lives of nearly 750,000 around the world (3300 from Saudi Arabia) [18, 19]. Moreover, the global overuse and misuse of antibiotics during the pandemic has dramatically increased, both in hospitals and in general due to increased self-medication for treating the symptoms of COVID-19 that may be confused with those of common cold. This increasing global consumption of antibiotics during the pandemic has caused intense fear of an acceleration in antimicrobial resistance [20–22].

In Saudi Arabia, although the laws governing community pharmacies demand authentic prescriptions (written or electronic) for dispensing prescription-only medications [23], antibiotics were commonly sold over the counter by these pharmacies [24, 25]. However, in 2018, the Saudi Ministry of Health launched a campaign to promote the implementation of the operational regulations for the pharmacy system, which led to a decrease in sales of over-the-counter antibiotics in community pharmacies [26]. Nonetheless, the adherence to these regulations during the COVID-19 outbreak has not been studied yet. Unfortunately, the level of public awareness among Saudi citizens regarding the misuse of antimicrobials and the resulting antimicrobial resistance is still low as reported by a recent survey [27].

The aim of this study was to investigate the over-the-counter sale of antibiotics by community pharmacies in Madinah, Saudi Arabia during the COVID-19 outbreak, and the quality of patient counseling offered by the pharmacists. In addition, the pharmacist triaging skills of patients with possible symptoms of the infection were documented.

## Methods

This cross-sectional, simulated-client study was conducted during July and August 2020, and the pharmacies were surveyed outside the lockdown times (i.e., during times when roaming across the city was allowed). The simulated-client technique was used to limit the Hawthorne Effect, i.e., possible behavioral changes by pharmacists if they were informed that they were being investigated.

### Simulated clients

Nine final-year pharmacy students from the College of Pharmacy, Taibah University, Madinah, Saudi Arabia, were trained by the researcher using a standard scenario and a simple form to be filled in after visiting each pharmacy. The researcher joined the students during the pilot test phase, and he also joined them randomly during the study period. Each pharmacy was visited by two students at the same time. One of them was responsible for the interaction with the pharmacists, and the other, who behaved as a separate customer, acted as an observer to assist the interacting student in filling in the survey form. This technique aided in minimizing the recall bias by the interacting student.

### Pharmacy selection

Although the number of community pharmacies in Madinah was 353 as reported by a recent study [28], it was difficult to include a large sample in this research. The difficulty was due to the various lock down periods and the risk of contracting the COVID-19 infection by the simulated clients due to the increased rate of infection at the time of the current research. Therefore, Eq. 1 was used to calculate the size of a randomly selected sample, where *n* is the sample size, *t*_*α*_ is 1.96 at confidence level of 95%, *p* is the response rate, *q* is the non-response rate (1 – *p*), and *e* is the accepted margin of error [29]. This method disregarded the population size and assumed a response rate *p* of 50% with a margin of error of 10%. Although the calculated sample size was 96, however, we were able to include 120 pharmacies. Simple random sampling was employed, where the list of the 353 registered pharmacies was coded and randomly scrambled using Microsoft Excel (Microsoft Corporation, Redmond, WA, USA), and the first 120 pharmacies (34%) were selected. All operating pharmacies were included. If a pharmacy was found closed, it was replaced by the next one from the remaining scrambled list.

1 $$n=\frac{t_{\alpha}^2\ast p\ast q}{e^2},$$

### Protocol for interaction with pharmacists

The simulated client waited until called by the pharmacist, greeted the pharmacist, and requested a pack of azithromycin 500 mg, vitamin C 1 g, and acetaminophen 500 mg. If the pharmacist asked about the purpose, the client said that it was for his mother who suffers from flu-like symptoms. If asked whether the patient had undergone the polymerase chain reaction (PCR) testing for COVID-19, the client would reply in the negative. Next, if the pharmacist requested a prescription for the antibiotic, the client would present the following demands in sequence, attempting to convince or embarrass the pharmacist into agreeing to sell an antibiotic without a prescription:

- Demand level 1: The client would say that he always buys it without a prescription.
- Demand level 2: If the pharmacist insisted on the prescription, the client would respond that he is a pharmacy student familiar with the use and misuse of antibiotics.
- Demand level 3: If the pharmacist continued to demand a prescription, the client would promise to bring a prescription later.
- Demand level 4: Despite this, if the pharmacist still refused to sell azithromycin without a prescription, the client would request amoxycillin 500 mg as an alternative. Amoxicillin has been found to be more widely sold over the counter in Saudi Arabia compared to other antibiotics, possibly because it is believed to be a mild antibiotic [25].
- Demand level 5: If the pharmacist continued to refuse, the client would ask him to suggest a hospital or a clinic from which he can obtain a prescription without his mother going.
- Demand level 6: Finally, the client would make a final attempt to win over the pharmacist and get the antibiotic.

Otherwise, if the pharmacist agreed to sell the antibiotic, his counseling behavior was observed by the client, who would later pretend that he forgot to bring his wallet and act like he was going home to bring it so that he can pay for the medicines. He would then thank the pharmacist and leave.

### Survey form

A simple form was filled in by each client immediately after leaving each pharmacy, assisted by the observer client, to overcome any recall bias. The first page contained the client’s name, the pharmacy’s code, name and location, and the date and time of the visit. These data were filled in by the simulated clients before entering the pharmacies to keep them focused on the interaction with the pharmacists. In addition, the sex and nationality of the pharmacist were also included in the first page.

The second page contained the same pharmacy code and all expected pharmacist responses, in addition to a checklist of the main queries asked during counseling such as the reason for using the antibiotic, presence of fever and other symptoms, body temperature reading, pregnancy and lactation, and the existence of allergies. The most important issue was whether the pharmacist asked about the presence of symptoms of COVID-19 (e.g., fever, cough, and loss of taste and smell) and whether the patient has undergone PCR testing. All possible pharmacist responses were included in the survey form as simple checkboxes, again to save time for the simulated clients and keep them focused on the interaction with the pharmacists, minimizing recall bias. The pharmacist’s recommendations were recorded and whether he/she advised the client to refer his mother to health facilities designated for COVID-19.

Finally, the first pages of the forms were collected by the leader of the simulated client group to be kept confidential, while other pages were handed to the researcher for analysis.

### The pilot test

The survey protocol was pilot tested on 18 pharmacies that were not among the study sample. These pharmacies were selected by convenience sampling, and each of them was visited by the researcher and two students. This way each student had a chance to directly interact with a pharmacist twice, as well as to observe another student’s interaction twice as well. The researcher and the observer student entered the pharmacies a minute after the interacting student as separate customers. This approach aided in the validation of the protocol, including easy completion of the survey form and overcoming recall bias.

### Data analysis

Simple descriptive analysis in the form of frequencies and ratios was employed.

### Ethical and safety considerations

Simulated clients were instructed to wear facial masks during pharmacy visits with the use of hand disinfectant before and after each visit. They were also instructed to maintain social distancing with 2 m between them and other customers. No personal information was gathered from the pharmacists, and information regarding the pharmacy was kept confidential.

## Results

In this simulated client study, 120 randomly selected community pharmacies in Madinah, Saudi Arabia were surveyed, of which seven were added in place of the pharmacies that were closed at the time of the visits. Of the surveyed pharmacies, 92 (76.7%) were belonging to chains while 28 (23.3%) were independent. All encountered pharmacists were Arabs, four of whom (3.3%) were Saudis of whom three were the only female pharmacists encountered in the survey.

It was found that 19 pharmacists (15.8%) opted to sell antibiotics without a prescription either instantly, or after the client stated that he is a pharmacy student, promised to bring the prescription later, or requested another antibiotic thought to be milder than the first one. In addition, 19 pharmacists (15.8%) who refused to sell an antibiotic over the counter, suggested a nearby private clinic that may issue a prescription upon paying a checkup fee without the need for bringing the patient. Surprisingly, one pharmacist (0.8%) referred the client to another pharmacy to obtain the antibiotic without a prescription. Table 1 summarizes the pharmacists’ responses to their clients’ requests to buy the antibiotic without a prescription.

**Table 1:** Pharmacists’ behavior when selling antibiotics without a prescription

Pharmacists’ responses	Number of pharmacists (out of 120) n (%)
Refused to sell without a prescription	81 (67.5)
Refused to sell without a prescription but suggested a nearby clinic that can issue antibiotic prescriptions	19 (15.8)
Refused to sell without a prescription but referred the client to another pharmacy that sells antibiotics over the counter	1 (0.8)
Agreed to sell azithromycin instantly	12 (10)
Agreed to sell azithromycin because the client said he is a pharmacy student	1 (0.8)
Agreed to sell azithromycin after client promised to bring the prescription later	1 (0.8)
Agreed to sell amoxicillin instantly instead	5 (4.2)
Total	120 (100)

As shown in Table 2, 29 pharmacists (24.2%) did not demonstrate any concern about the possibility that the patient may be infected with COVID-19 instead of com-mon influenza. Other pharmacists (39.2–55%) expressed their concerns but provided unequal recommendations regarding the PCR test, COVID-19 symptoms, patient isolation, and seeking medical attention for the patient.

**Table 2:** Pharmacists’ concerns and recommendations regarding COVID-19

Pharmacists’ responses	Number of pharmacists (out of 120) n (%)^a^
Asked if the patient had undergone the PCR test for COVID-19	66 (55)
Asked about COVID-19 symptoms	47 (39.2)
Recommended performing the PCR test	59 (49.2)
Recommended seeking medical attention	61 (50.8)
Recommended isolating the patient	53 (44.2)
No response	29 (24.2)

^a^ Participants are 120; however, they gave mixed responses

*Abbreviation*: *PCR* polymerase chain reaction

Finally, regarding the counseling behavior of pharmacists who agreed to sell the antibiotics without a prescription (*n* = 19), it was shown that 12 of them (63.2%) did not offer any counseling according to the patient’s identity, their condition, whether they were pregnant or lactating, had existing allergies, and the frequency and duration of treatment. The remaining pharmacists, however, offered varying levels of counseling as shown in Table 3.

**Table 3:** Pharmacists’ behavior when selling antibiotics without a prescription

Pharmacists’ responses	Number of pharmacists (out of 19) n (%)^a^
Asked who will use the antibiotic	4 (21.1)
Asked about the purpose of using the antibiotic	5 (26.3)
Asked if the patient is pregnant	1 (5.3)
Asked if the patient is breastfeeding	1 (5.3)
Asked if the patient has allergies	1 (5.3)
Explained the schedule and duration of treatment	1 (5.3)
No response	12 (63.2)

^a^ Participants are 19; however, they gave mixed responses

## Discussion

A recent study reported a low level of preparedness among Saudi community pharmacies during the COVID-19 outbreak in terms of the availability of good quality face masks and hand disinfectants, as well as adherence to preventive measures and effective contribution to increasing community awareness of the pandemic [28]. The current study, however, focused on another critical issue. To our knowledge, this is the first study that surveyed the nonprescribed sale of antibiotics by community pharmacies during the COVID-19 outbreak in Saudi Arabia, which is a violation of the system for pharmacy practice in this country [23]. In addition, instead of performing convenience sampling as adopted in that recent study, the current study employed random sampling of almost one third of the community pharmacies in Madinah, Saudi Arabia because it was conducted when roaming across the city was permitted within certain times during the lockdown period.

Although the sale of antibiotics without a prescription in community pharmacies in Saudi Arabia had decreased significantly from that reported in previous studies published between 2011 and 2013 [24, 25], this malpractice still takes place, at least in part. In fact, the current study’s percentage of pharmacies selling antibiotics over the counter during the COVID-19 outbreak in Saudi Arabia is greater than that reported in a recent study conducted in 2018 (15.8 and 12.5%, respectively) [26]. What may make the situation worse is that 16.7% of the pharmacists who refused to commit such malpractices, referred the clients to clinics from where they can easily obtain prescriptions, or to other pharmacies that sell the antibiotics over the counter. The observed increase in the nonprescribed sale of antibiotics demands rapid intervention from relevant authorities such as the Saudi Food and Drug Authority and the Ministry of Health, given the risks involved in the misuse of antibiotics, represented by an in-crease in bacterial resistance, especially during this pandemic [20–22].

One of the interesting results from this survey is that community pharmacists generally failed to triage patients who should have been referred to facilities designated for diagnosing COVID-19 infection through the PCR test. Community pharmacists are expected to play a pivotal role during disasters as frontline healthcare providers. Therefore, they should be well prepared and guided to triage patients and offer suitable patient education [30]. Unfortunately, the current survey showed that only 55% of them asked whether the patient had tested for COVID-19 using PCR, 39% asked about the symptoms, and 44% recommended patient isolation. Moreover, around 50% of the pharmacists recommended the PCR test and seeking medical attention. Alarmingly, 24% of pharmacists were completely careless about the possibility that the patient might have caught the infection. These findings indicate the unpreparedness of community pharmacies in Saudi Arabia for the pandemic as reported earlier [28].

Although only 15.8% of pharmacists in this study violated the laws of pharmacy practice by selling antibiotics over the counter, the level of patient counseling provided was extremely unacceptable. Around 63% of them were completely ignorant about the provision of basic information to the customers (such as the frequency and duration of treatment) and asking about who will use the antibiotic, for what purpose, or even whether the patient has allergies to certain medications, is pregnant, or is breastfeeding. This finding goes in line with the results of a previous study, which showed that the level of providing basic information to customers by community pharmacists in Saudi Arabia was generally low [31].

Finally, it is worth mentioning that Saudi pharmacists, although their number in this study is very low compared with non-Saudis (4 and 116, respectively), were not among the violators of the system. In addition, their counseling behavior and role in triaging and increasing community awareness regarding the COVID-19 pandemic was remarkable. However, their low percentage (3.3%) puts the issue of localizing this profession at stake. In 2020, the Saudi Ministry of Labor and Social Development (currently renamed as the Ministry of Human Resources and Social Development) reported that the localization ratio of Saudi pharmacists in the private sector was only 13% [32]. Moreover, this report did not indicate the percentage of those working in community pharmacies. This, in turn, necessitates conducting studies that investigate the reasons for the reluctance of Saudi pharmacists to work in this vital sector.

### Limitations

Due to the nature of this simulated-patient study, it was not possible to retrieve important predictors that might influence the nonprescribed sale of antibiotics by community pharmacies (e.g., the pharmacists’ sociodemographic data, age, acquired academic degree, number of staff per shift, pharmacy turnover, and the sociodemographic status of the customers served). Nonetheless, this observational study aimed to investigate the spontaneous responses of the community pharmacists and did not intend to distinguish the causality of these responses.

Moreover, despite the training the simulated clients received in addition to the researcher accompanying them during the pilot test, the fact that they were nine different data collectors may have influenced the consistency of the performed role playing and the recorded observations. This may have resulted in a lower, or even higher rate of acceptance to sell nonprescribed antibiotics by the community pharmacists.

Finally, this study was limited by its relatively small scale because it was conducted in only one main city of Saudi Arabia. Although several pharmacy chains in this city are spread throughout the country and are expected to follow similar patterns of pharmacy practice, differences are expected among these chains in addition to the other independent pharmacies. Therefore, future studies are highly recommended. These studies may include a representative sample of community pharmacies in different regions, aiming to identify the predictors associated with over-the-counter sale of antibiotics.

## Conclusions

The risk of bacterial resistance is exacerbated by the misuse and overuse of antimicrobials. The malpractice of selling antibiotics without a prescription by community pharmacies is still prevalent in Saudi Arabia, especially during the COVID-19 pandemic. Community pharmacists in this country have failed to triage and counsel customers regarding the pandemic and provided very less information regarding the use of antibiotics. Despite the small number of Saudi pharmacists in this study, they have demonstrated full adherence to the practice regulations, and their provision of necessary patient education regarding the COVID-19 pandemic was optimal. Our findings serve as a cautionary note to community pharmacists to improve their quality of service. Further follow-up studies are recommended, and the health authorities in Saudi Arabia must adopt stronger enforcement procedures to ensure the adherence of community pharmacies to the regulations. Finally, Saudi pharmacists should be encouraged to work in community pharmacies by offering them more incentives.

## Data Availability

The data that support the findings of this study are available on reasonable request from the corresponding author. The data are not publicly available to maintain the confidentiality of research participants.
